# Development and Experimental Validation of a Numerical Tool for Structural Health and Usage Monitoring Systems Based on Chirped Grating Sensors

**DOI:** 10.3390/s150101321

**Published:** 2015-01-12

**Authors:** Paolo Bettini, Erika Guerreschi, Giuseppe Sala

**Affiliations:** Department of Aerospace Science and Technology, Politecnico di Milano, 20156 Milan, Italy; E-Mails: erika.guerreschi@polimi.it (E.G); giuseppe.sala@polimi.it (G.S.)

**Keywords:** structural health monitoring, Chirped Fibre Bragg Gratings, Draw Tower Grating Array, strain reconstruction

## Abstract

The interest of the aerospace industries in structural health and usage monitoring systems is continuously increasing. Among the techniques available in literature those based on Fibre Bragg Grating sensors are much promising thanks to their peculiarities. Different Chirped Bragg Grating sensor configurations have been investigated in this paper. Starting from a numerical model capable of simulating the spectral response of a grating subjected to a generic strain profile (direct problem), a new code has been developed, allowing strain reconstruction from the experimental validation of the program, carried out through different loading cases applied on a chirped grating. The wavelength of the reflection spectrum for a chirped FBG has a one-to-one correspondence to the position along the gauge section, thus allowing strain reconstruction over the entire sensor length. Tests conducted on chirped FBGs also evidenced their potential for SHM applications, if coupled with appropriate numerical strain reconstructions tools. Finally, a new class of sensors—Draw Tower Grating arrays—has been studied. These sensors are applicable to distributed sensing and load reconstruction over large structures, thanks to their greater length. Three configurations have been evaluated, having different spatial and spectral characteristics, in order to explore possible applications of such sensors to SHM systems.

## 1. Introduction

Nowadays, structural health monitoring represents one of the major concerns for modern aeronautical structure maintenance and aircraft fleet management [1]. As a matter of fact, the progressive ageing of the aircraft fleet can make questionable their durability, affordability and profitability, as well as the conventional preventive maintenance philosophy, based on a scheduled-based approach and non-destructive inspection techniques. Such uncertainties become even more alarming when the structures are made of composite materials [2]. On the contrary, the ageing of modern damage-tolerant composites aeronautical structures is better managed through a predictive maintenance philosophy, sometimes based on condition-based approaches and requiring integration of a network of sensors, actuators and detection algorithms. The structures become smart structures and the philosophy is called structural health monitoring (SHM), usually implemented in modern aircraft through health and usage monitoring systems (HUMS). Such methodology is very promising and is going to be adopted by most modern aircraft, provided some major concerns can be successfully tackled, such as sensor and actuator integration, data processing and storage, noise and false signals filtering, environmental and variable operational conditions management [3,4].

The design of an efficient health monitoring system is a complex and multidisciplinary operation, strongly dependent on monitoring strategy and type of host structure. However, three major elements are present in every HUMS:

- Monitoring System: Sensors, networks and instruments used for the acquisition of significant quantities (strain, temperature);
- Diagnosis System: Central processor and software for analyzing measured data in order to evaluate structural health;
- Prognosis System: Software for predicting residual life of the structure on the basis of damage entity, propagation and load history.

In the present paper the first two aspects are investigated, aiming at the realization of a complete health monitoring system based on Chirped Fibre Bragg Gratings (CFBGs). Fibre optic sensors are very promising thanks to their peculiarities in terms of shape and size, low invasivity if embedded in composite laminates, immunity to electromagnetic fields, multiplexing capabilities, resistance to extreme environments and their capabilities of measuring different physical quantities [5]. Moreover, with respect to the standard uniform FBG, which can transduce only the average strain on its total length, a chirped one is able to provide information about the strain distribution profile along the grating itself. In fact, having a variable grating period, this kind of sensors has a one-to-one correspondence between reflection spectrum wavelength and position on the sensor [6–8]. Thus, the analysis of the shape and position changes of the reflection spectrum permits to retrieve the applied strain, making chirped gratings ideal for distributed sensing over short lengths (< 30–50 mm is the maximum length of the chirped available on the market).

In the first part of the work a code for the numerical simulation of the reflection spectrum has been developed and configured with an optimization algorithm. The resulting code is capable of reconstructing the strain profile acting on the chirped sensor given its experimental reflection spectrum. The code has successively been tested on a chirped grating subjected to different known strain profiles.

Finally, a recent novel optical sensor known as a Draw Tower Grating (DTG) array has also been taken into consideration. These arrays are produced by means of an innovative technique whereby gratings are inscribed directly on the draw tower, before coating deposition. This procedure avoids fibre stripping and recoating, considerably increasing sensor tensile strength and reliability [9–11]. As a result DTGs also have a lower unit cost if compared with conventional arrays. Furthermore DTG arrays' spectral characteristics can easily be tuned in order to obtain the most various spectral shapes and sensor responses. For instance, spatial continuum arrays (without gaps between the gratings) can exhibit non continuum spectra as well spatial non continuum arrays can also associated to the continuum spectra. Doing so, sensors completely similar to the chirped ones can be obtained with higher dimensions (1–10 m long arrays can be easily produced at relative moderate costs). Three array configurations have been tested in order to identify the most suitable one(s) for strain monitoring applications.

## 2. Developed Strain Reconstruction Technique: The *FBG Strain* Tool

The first step in the development of a strain reconstruction tool is the implementation of an algorithm simulating the reflection spectrum of a sensor subject to an arbitrary strain profile. Such an algorithm can then be coupled to an optimization procedure which finds the strain profile minimizing the difference between simulated and experimental spectra [12–14]. Common grating simulation techniques are based on the Coupled Mode Theory (CMT), a simplified theory derived from Maxwell's equations [15] which describes light propagation in an optical guide. Taking a reference system having *z* coincident with the fibre axis and (*x*, *y*) on the perpendicular plane, electrical field propagating inside the fibre core can be described as the superposition of j modes [16]:

(1) $${\text{E}}_{t}\left(x,y,z,t\right)=\sum_{j}\left[A_{j}\left(z\right){\text{e}}^{i\beta_{j}z}+B_{j}\left(z\right)e^{-i\beta_{j}z}\right]e_{jt}\left(x,y\right)e^{-i\omega t}$$

where *A_j_*(*z*) and *B_j_*(*z*) are the amplitudes of the modes propagating in the +*z* (transmission) and −*z* (reflection) directions respectively. Transverse field distribution for each mode is described by ***e****_jt_*(*x, y*) and *e*^−^*^iωt^* indicates time oscillation. If the guide is unperturbed counter-propagating modes are orthogonal. If a discontinuity, such as a Bragg grating, is present, mode amplitudes (and therefore their field intensities) are coupled, and their spatial distribution along *z* is described by the following equations:

(2) $$\{\begin{array}{c} \frac{dA_{j}}{dz}=i\sum_{k}A_{k}\left(K_{kj}^{t}+K_{kj}^{z}\right)e^{i\left(\beta_{k}-\beta_{j}\right)z}+i\sum_{k}B_{k}\left(K_{kj}^{t}-K_{kj}^{z}\right)e^{-i\left(\beta_{k}+\beta_{j}\right)z}\\ \frac{dB_{j}}{dz}=-i\sum_{k}A_{k}\left(K_{kj}^{t}-K_{kj}^{z}\right)e^{i\left(\beta_{k}+\beta_{j}\right)z}-i\sum_{k}B_{k}\left(K_{kj}^{t}+K_{kj}^{z}\right)e^{-i\left(\beta_{k}-\beta_{j}\right)z} \end{array}$$

where $K_{kj}^{t}$ and $K_{kj}^{z}$ are the transverse and longitudinal coupling coefficients between modes *j* and *k*. Typically $K_{kj}^{z}$ is smaller than $K_{kj}^{t}$ and therefore is usually neglected. Optical fibres commonly used for Bragg grating inscription are single-mode fibres, having only two identical counter-propagating modes. In the latter case, Equation (2) can be further simplified [16], yielding:

(3) $$\{\begin{array}{c} \frac{dR\left(z\right)}{dz}=i\hat{\sigma }R\left(z\right)+\text{ikS}\left(z\right)\\ \frac{dS\left(z\right)}{dz}=-i\hat{\sigma }S\left(z\right)-ik^{*}R\left(z\right) \end{array}$$

where $R\left(z\right)=A\left(z\right)e^{i\delta z-\frac{\phi }{2}}$ and $S\left(z\right)=B\left(z\right)e^{-i\delta z+\frac{\phi }{2}}$ are the transmission and reflection modes respectively, *k* and k* are coupling coefficients, while *σ̂* is a self-coupling coefficient defined as:

(4) $$\hat{\sigma }=\delta +\sigma -\frac{1}{2}\frac{d\phi }{dz}$$

Detuning *δ*, which is *z*-independent, is defined as the difference between the mode propagation constant at a given wavelength and the “design” propagation constant:

(5) $$\delta =\beta -\beta_{B}=2\pi n_{\text{eff}}\left(\frac{1}{\lambda }-\frac{1}{\lambda_{B}}\right)$$

where *n_eff_* and *λ_B_* are the effective refractive index and the Bragg's wavelength respectively.

The derivative $\frac{d\phi }{dz}$ describes a possible grating chirp, which is *z*-dependent. For a single mode reflection grating coefficients σ, k, k* are defined as follows:

(6) $$\sigma =\frac{2\pi }{\lambda }{\bar{\delta n}}_{\text{eff}}\;k=k^{*}=\frac{\pi }{\lambda }\nu {\bar{\delta n}}_{\text{eff}}$$

${\bar{\delta n}}_{\text{eff}}$ is the background refractive index change while *ν* is the the Poisson's coefficient.

**Figure 1.**
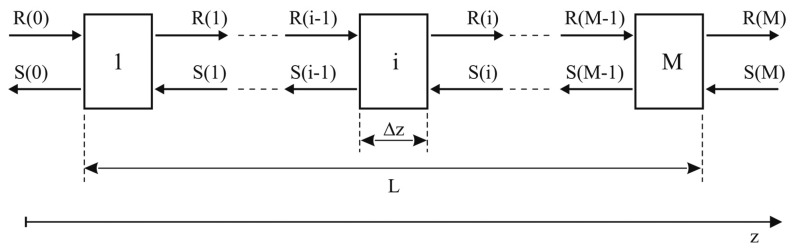
Transfer Matrix Approach—Grating modelled as a sequence of uniform sub-gratings.

If the grating is uniform, both grating period and refractive index change are constant along the fiber axis *z*. Equations (2) and (3) thus become coupled ordinary differential equations with constant coefficients, for which close-form solutions can be obtained, given the appropriate boundary conditions. In the more general case of a non-uniform grating, whose period or effective refractive index variation are a function of the *z* coordinate, numerical approaches should be used in order to obtain a relationship between S and R at the grating extremities. A first possible approach consists in the numerical resolution of Equations (2) and (3), typically by means of a Runge-Kutta scheme. However, even if an appropriate numerical scheme is applied, this method is slow if compared with other possible approaches having the same accuracy [17,18]. An alternative, widely used and relatively fast method is based on the piecewise-uniform approximation of the coefficients *σ̂*, *k* and k*. This method is known as the Transfer Matrix Method (TMM) and is based on the division of the non-uniform grating into M uniform gratings (Figure 1), each having constant properties corresponding to a piecewise-uniform approximation of the chirp and apodization profiles of the original grating. Since the analytical solution of Equations (2) and (3) is known, for each subsection it can be written, with reference to Figure 1:

(7) $$\left[\begin{array}{c} R_{i-1}\\ S_{i-1} \end{array}\right]={\text{F}}_{i}\left[\begin{array}{c} R_{i}\\ S_{i} \end{array}\right]$$

where ***F****_i_* is the transfer matrix for the *i*-th section:

(8) $${\text{F}}_{i}=\left[\begin{array}{cc} \cosh\left(\gamma_{B}\Delta z\right)-\frac{i\hat{\sigma }}{\gamma_{B}}\sinh\left(\gamma_{B}\Delta z\right) & -\frac{ik}{\gamma_{B}}\sinh\left(\gamma_{B}\Delta z\right)\\ \frac{ik}{\gamma_{B}}\sinh\left(\gamma_{B}\Delta z\right) & \cosh\left(\gamma_{B}\Delta z\right)+\frac{i\hat{\sigma }}{\gamma_{B}}\sinh\left(\gamma_{B}\Delta z\right) \end{array}\right]$$

Coefficient *γ_B_* is defined as:

(9) $$\gamma_{B}=\sqrt{k^{2}-{\hat{\sigma }}^{2}}$$

Once ***F****_i_* is calculated for every sub-grating the transfer matrix for the entire grating ***F*** can be obtained multiplying the subsection transfer matrices:

(10) $$\left[\begin{array}{c} R_{0}\\ S_{0} \end{array}\right]={\text{F}}_{1}{\text{F}}_{2}\cdots {\text{F}}_{i}\cdots {\text{F}}_{M}\left[\begin{array}{c} R_{M}\\ S_{M} \end{array}\right]=\text{F}\left[\begin{array}{c} R_{M}\\ S_{M} \end{array}\right]$$

Finally, reflection and transmission amplitudes *r*(*λ*) and *t*(*λ*) can be calculated for every wavelength by assigning the boundary conditions *R_o_* = 1 and *S_M_* = 0:

(11) $$r\left(\lambda \right)=\frac{S_{0}}{R_{0}}=\frac{F_{21}}{F_{11}}\;t\left(\lambda \right)=\frac{R_{M}}{R_{0}}=\frac{1}{F_{11}}$$

and consequently reflectivity *R* = *r*^2^ and transmission coefficient *T* = *t*^2^.

After having implemented the Transfer Matrix Method in a Matlab code, an optimization algorithm has been adopted for creating an effective tool able to identify the strain profile acting on the sensor given its experimental spectrum.

**Figure 2.**
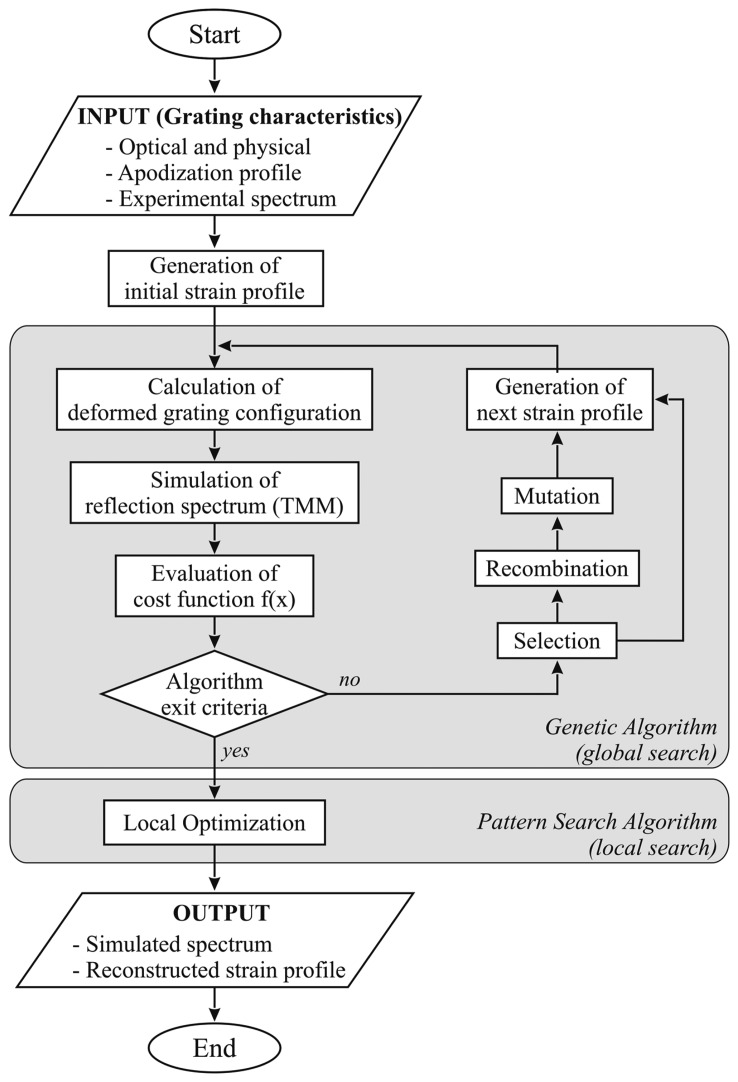
Strain reconstruction technique.

In particular, a hybrid genetic algorithm has been used. This class of algorithms is based on the evolution of a population of individuals. Each individual represents a possible solution of the optimization problem. Evolution is obtained by means of selection, reproduction (crossover) and mutation, mimicking natural evolution [12]. This process is illustrated in Figure 2.

The cost function to be minimized is defined as follows, and accounts for the major variables influencing spectral shape:

(12) $$f\left(\text{x}\right)=W_{\text{int}}\frac{\left\|r_{\text{ref}}-r_{\text{sim}}\right\|}{\left\|r_{\text{ref}}\right\|}+W_{\lambda }\frac{\left\|\lambda_{B_{\text{ref}}}-\lambda_{B_{\text{sim}}}\right\|}{{\text{FWHM}}_{\text{ref}}}+W_{w}\frac{\left\|{\text{FWHM}}_{\text{ref}}-{\text{FWHM}}_{\text{sim}}\right\|}{\left\|{\text{FWHM}}_{\text{ref}}\right\|}$$

The first term is proportional to the root of squared errors between the experimental (reference) and simulated spectra, for every wavelength considered. The second one is proportional to the difference between reference and simulated Bragg wavelengths. This latter term should be considered only if small variations in the spectral shape occur, caused by uniform or linear strain profiles. Finally, the last term is relative to the spectrum Full Width at Half Maximum. Each term is adequately normalized and weighted. The values of the strain in *N* points, named *control points*, on the sensor length represent the optimization variables ***x***.

In order to improve convergence time, genetic algorithm is used only in the first phase (global search) of the search process, in order to efficiently sample the space search, eventually approximating the global minimum [19]. The terminal part of the search procedure (local search) is carried out via a pattern search algorithm, a relatively fast gradient-free search method [20].

For each trial solution, represented by a vector containing strain values at control points, sensor reflection spectrum is simulated using the TMM-based program and compared with the real sensor spectrum, obtained through an optical spectrum analysis.

The minimum of the cost function corresponds to the set of strain values which minimizes the difference between the experimental and numerical spectrum, and therefore better approaches the real strain profile applied to the sensor.

Due to small imperfections in the inscription process as well as inevitable experimental errors, the theoretical reflection spectrum, obtained through TMM-based simulation, presents important shape difference from the experimental one. In order to eliminate this discrepancy, an additional tool has then been developed, using an approach similar to the one described above. The difference is that, this time, the optimization procedure is aimed at the identification of the apodization profile of the real sensor. Problem variables are refractive index modulation at a number of control points, and the cost function is defined in analogy with the one described in Equation (12). The reference reflection spectrum is the one relative to the unloaded sensor, the only difference with the numerical spectrum being the apodization profile.

**Figure 3.**
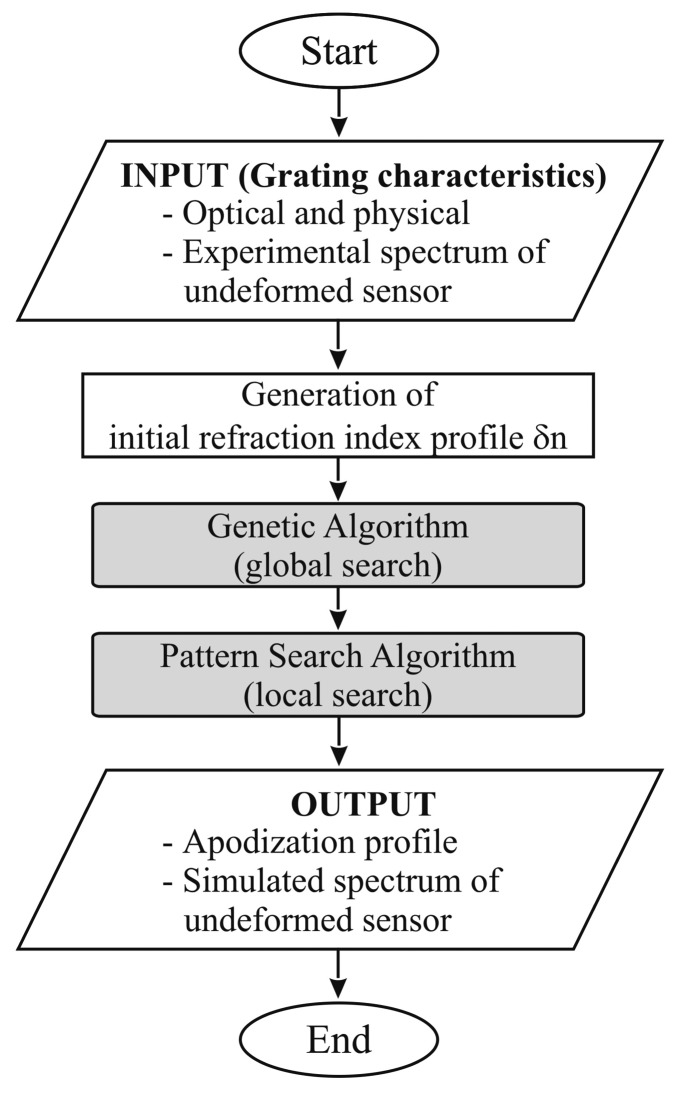
Apodization profile identification.

Once the apodization profile that minimizes the differences between numerical and experimental undeformed spectra, it should be used to correctly simulate sensor response in the strain reconstruction process. Apodization identification procedure is described in Figure 3.

Since each tentative strain/apodization requires a cost function evaluation, and therefore the simulation of the corresponding reflection spectrum, the entire process requires a considerable amount of time. In fact, the computation time depends on a large number of variables including setting parameters of the optimization algorithms (such as control points number, exit criteria, selection, recombination and mutation parameters) as well those corresponding to the spatial and spectral mesh discretization (such as sub-gratings and fringes numbers, wavelength resolution). For this reason the aforesaid procedure is not applicable to real-time strain monitoring.

## 3. Experimental Activities

### 3.1. Chirped Sensors

Several tests have been carried out on a CFBG, with the aim of both validating the strain reconstruction tool and evaluating the practical use of such class of sensors. Physical and optical characteristics of the CFBG used for the tests are summarized in the Table 1.

The choice of the test cases has been strongly constrained by the need of exactly knowing the strain profile applied to the sensor. Given the limited dimensions of tested sensors, uniform and linear strain profile are the only practically applicable. Three case studies have been considered:

- Uniform strain—tensile test;
- Linear strain—3-point bending test;
- Linear strain with gradient change—3-point bending test.

**Figure 4.**
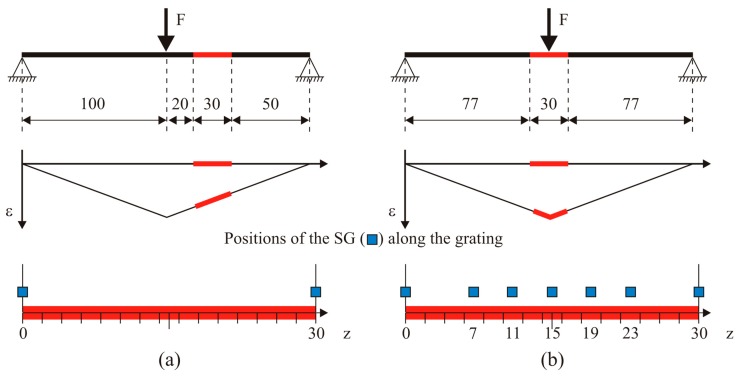
Three-point bending tests performed on the chirped grating: linear strain (**a**) and linear strain with gradient change (**b**). The position of the chirped is highlighted by red lines while blue markers identify positions of SG. Dimensions are in mm.

The second and third case can be obtained by changing the relative position between the sensor and the loading pins (see Figure 4). Sensors have been glued on different thin specimens made of ERGAL 7075. All the specimens were instrumented by strain gauges (SGs) in order to experimentally retrieve the applied strain profile. The geometrical characteristics of the specimens are reported in the Table 2 while sensors positions are illustrated in the Figure 4.

For each test study, several loading levels have been applied, with increasing strain magnitude to test repeatability and robustness of the strain reconstruction tool. Uniform strain levels were 500, 1000 and 1500 με. As for the linear case, maximum strain levels ranged from 1000 to 3500 με, with a step of 500 με. Due to small strain difference achieved between the centre and the extremities of the grating, which is not very significant for validation of the developed tool, in the third case only higher levels (2000 με and 2500 με) were applied.

**Figure 5.**
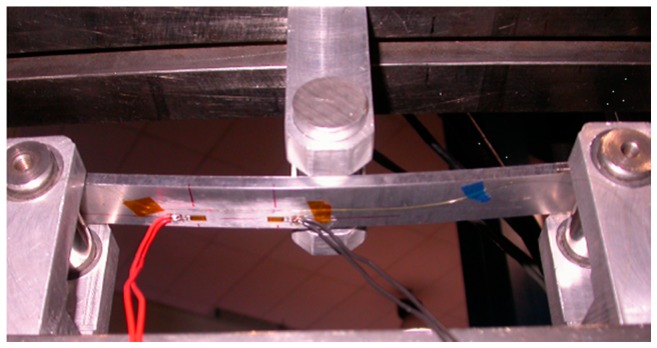
Experimental setup of the linear strain test case performed in 3-point bending configuration on chirped sensor.

Figure 5 shows the linear strain profile applied in 3-point bending. It can be seen that grating and strain gauges are located on the lower side of the specimen in order to be subject only to positive strain.

### 3.2. Draw Tower Grating Arrays

As mentioned in the introduction, a recent printing technique referred to as Draw Tower Gratings [21] gives the possibility to achieve arrays with several advantages with respect to traditional ones. One of the most interesting features of this class of sensors is the possibility of obtaining the most different combinations of spectral and spatial characteristics. In order to identify which combination is the most suitable for strain reconstruction applications, three arrays have been examined. Every array is 100 mm long and composed of 10 uniform FBG sensors:

- SPAtial Continuity and SPEctral Discontinuity (*SpaC/SpeD*): Each sensor is 10 mm long, no separation exists between two adjacent gratings. Sensors wavelengths are separated by ∼1 nm;
- SPAtial Continuity and SPEctral Continuity (*SpaC/SpeC*): Each sensor is 10 mm long, no separation exists between two adjacent gratings. Sensors wavelengths are separated by ∼0.1 nm, that is the FWHM of a single peak. Resulting spectrum is continuous, with local maxima corresponding to each FBG Bragg wavelength;
- SPatial Discontinuity and SPEctral Continuity (*SpaD/SpeC*): Each sensor is 3 mm long, 7 mm separation between two adjacent gratings. Wavelength separation is < FWHM, originating a continuous reflection spectrum. No local maxima are present, given the greatest width of the individual peaks.

**Figure 6.**
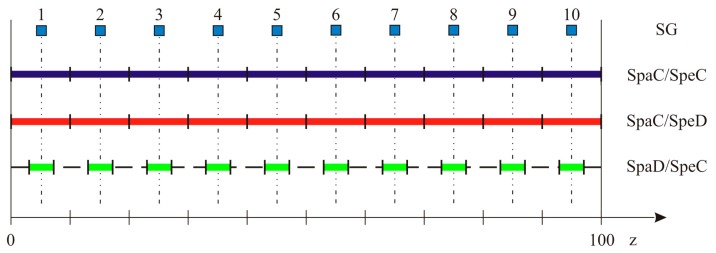
Arrays configurations and relative positions of strain gauges. SG are placed aligned with the center of each individual uniform grating.

**Figure 7.**
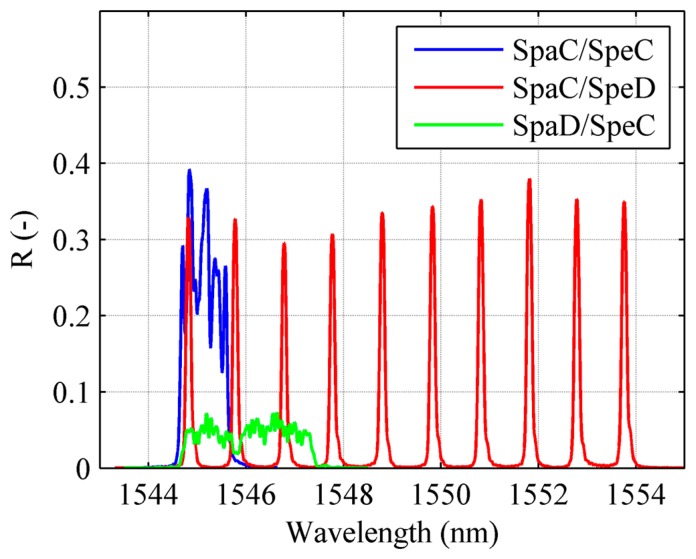
Experimental reflection spectrum for the three different configurations of adopted DTG arrays.

Spectral discontinuity allows individual peak tracking, and consequently real-time monitoring. On the other hand, a sufficient wavelength separation between individual peaks has to be guarantee in order to prevent superposition of two or more peaks (wavelength division multiplexing), thus limiting the number of single FBG which can be inscribed on the fibre. Besides this, peak tracking gives no information about strain gradients on the sensor. Spectral continuity generates narrower spectra, but requires spectral analysis and a numerical strain reconstruction technique as the one described in Section 2, making it impossible to perform real-time monitoring. Arrays configurations are illustrated in the scheme of Figure 6 while the corresponding reflection spectrum are reported in the Figure 7.

The main characteristics of the DTG arrays are summarized in the Table 3.

**Figure 8.**
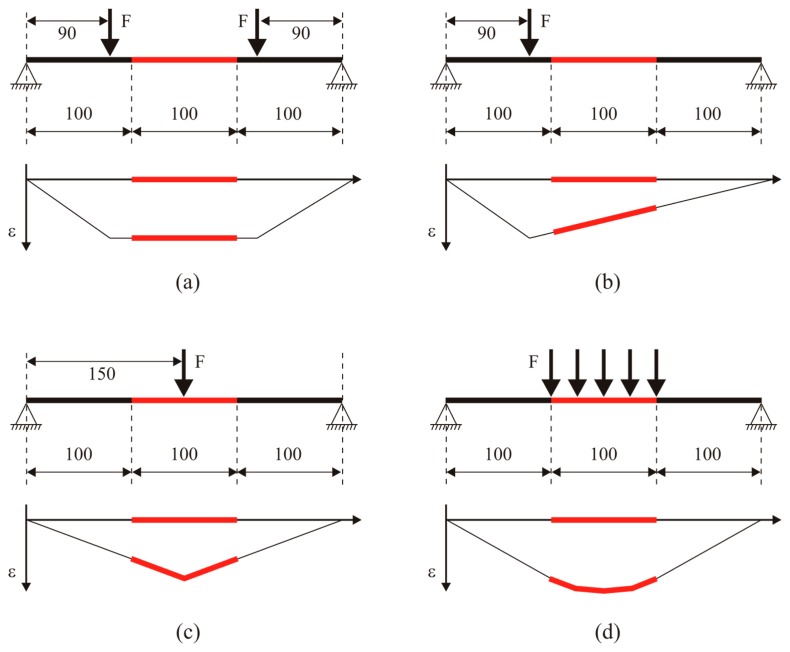
Case studies applied on the DTG arrays: uniform strain (**a**), linear strain (**b**), linear strain with gradient change (**c**) and quasi-quadratic strain profile (**d**). The position of the DTG arrays is highlighted by red lines while blue markers identify positions of SG.

In analogy with the chirped grating case, several tests have been carried out, in order to obtain different strain profiles on the sensor. The greater length of the DTG arrays with respect to the chirped sensor allowed more complicated strain profiles to be applied. Four case studies have been considered (see Figure 8):

- Uniform strain—4-point bending test;
- Linear strain—3-point bending test;
- Linear strain with gradient change (“triangular” strain profile)—3-point bending test;
- Quasi-quadratic strain—simply supported beam with quasi-distributed load.

**Figure 9.**
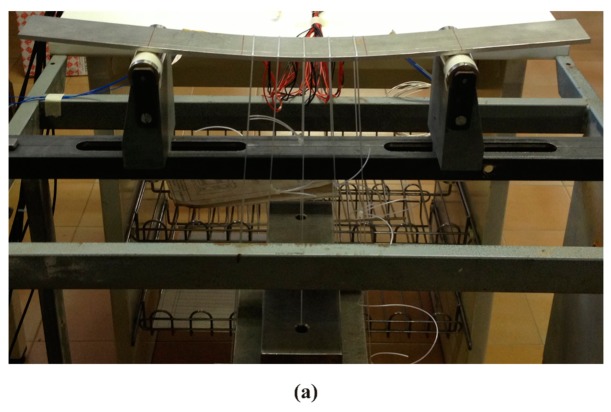

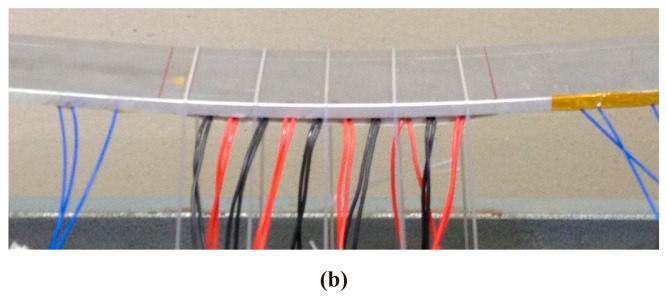
Experimental setup of the quasi-quadratic strain test case performed on DTG arrays (**a**) and view of the loading zone (**b**).

All DTG arrays have been glued on the same specimen made of ERGAL 7075. Specimen characteristics are reported in the Table 4. In order to guarantee their correct positioning, DTG arrays have been placed into three parallel slight V-grooves realized on the lower surface of the specimen. Load is applied via several conveniently placed dead-weights hanging from the specimen (Figure 9).

## 4. Results and Discussion

### 4.1. Chirped

On the base of the theory presented in Section 2, the CFBG has been modelled as a sequence of M uniform gratings for which analytical solution can be easily calculated. In particular the 30 mm long chirped has been divided into 565 sub-gratings made of about 100 fringes each one. A spectral domain of 80 nm centred at 1550 nm is discretized in 400 points resulting in a spectral resolution of 0.2 nm.

The number of fringes for each sub-grating as well the spectral resolution are the most important parameters of the model and the correct choice of them is fundamental for obtaining good simulations. In fact, low values of these don't allow to correctly reconstruct the strain profile while, at the opposite, too high values increase too much the time of genetic algorithm.

**Figure 10.**
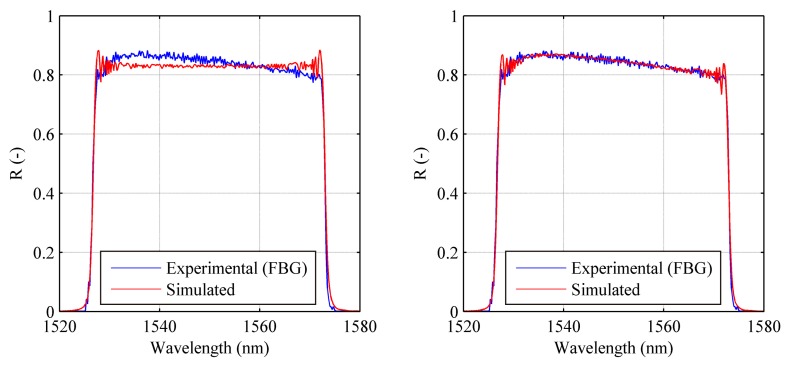
Chirped sensor: comparison between experimental and numerical spectra before (**left**) and after (**right**) apodization profile identification.

At first, identification of the sensor apodization profile was done as reported in the scheme of Figure 3. The result of this operation is reported in Figure 10, where the reflection spectrum of the unloaded sensor is compared with numerical spectra before and after apodization profile identification. It is worth noting that without this preliminary phase it would have been impossible to perform a strain reconstruction based on spectra comparison since numerical (theoretical) and experimental spectrum shapes are completely different. Apodization profile identification has been carried out by using seven control points distributed along the spectrum with a higher concentration at the edges.

**Figure 11.**
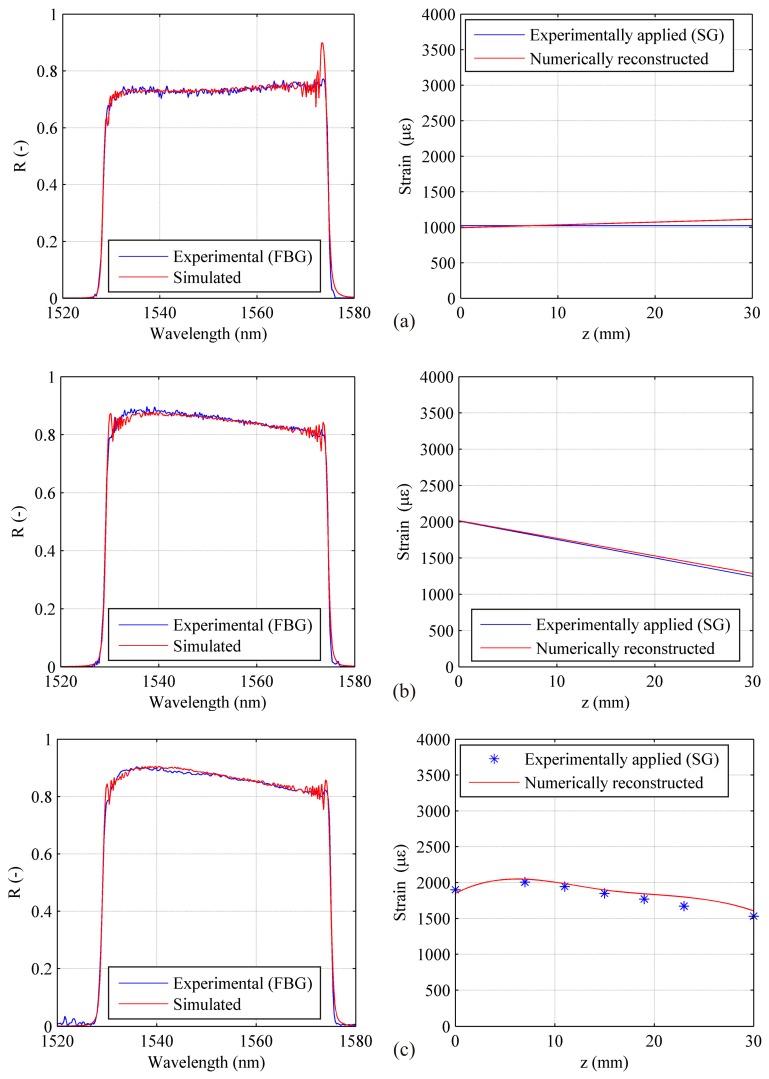
Chirped sensor: numerically reconstructed strain profile compared with the experimentally applied one (**right**) and corresponding spectra (**left**). Uniform strain test case at 1000 με (**a**); linear strain test case at a maximum level of 2000 με (**b**); Linear strain with gradient change test case at a maximum level of 2000 με (**c**).

Once the apodization profile is known, it can be used to correctly simulate the experimental reflection spectrum, and therefore to perform strain reconstruction. Initially in the first two test cases (uniform and linear strain), the strain was simulated using two control points corresponding to a linear approximation of applied strain, while in the third case the strain with gradient change was obtained using five control points in order to achieve a better strain trend. A single strain level is reported for all the test cases in the following Figure 11.

Strain gauges measurements are in agreement with beam theory confirming that can be assumed as reference for correlations with reconstructed strain profiles. Based on SG values, relative errors have been calculated by using the follows definition:

(13) $$e_{r}=\frac{{ɛ}_{r}-{ɛ}_{a}}{{ɛ}_{a}}100$$

where *ε_r_* is the reconstructed strain value and *ε_a_* is the corresponding applied one.

A maximum relative error of 5.8% was obtained at 500 με level for uniform strain tests (minimum of 1.3% at 1500 με). As for the linear case, maximum relative error occurred at the 1000 με case, with a value of 8.2% (minimum 4.9% at 3500 με). Finally, in the case of linear strain with gradient change the maximum relative error occurred at 2000 με was of 134 με.

The shape of the strain profile highlight an error occurred in the specimen positioning for this case. Nevertheless, SG and CFBG trend are consistent with each other. The computational time for full cycle analyses has been of about 40–80 min depending on the parameter setup.

**Figure 12.**
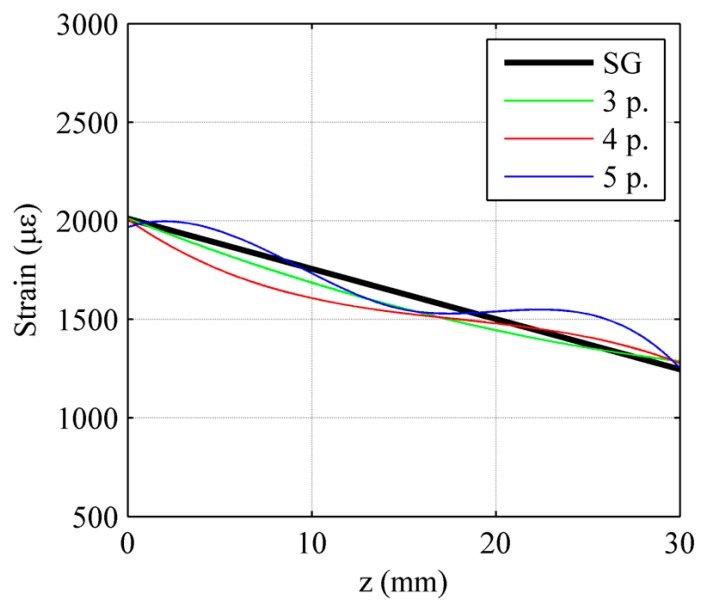
Chirped sensor: strain reconstruction using multiple control points and comparison with measured strain for the linear strain test case.

Subsequently influence of the number of control points has been investigated, both for the uniform and linear case. For three reference test cases (1500 με for the uniform strain case and 2000 με maximum strain for the linear one), identification has been carried out with an increasing number of control points. Figure 12 shows several profiles obtained using multiple control points for the linear case, showing an overall good approximation of the real strain profile.

It should be noted, despite the reconstructed strain profiles exhibit rather irregular trend, strain values at the sensor edges show a very low dispersion. This is due to a high sensitivity of the numerical code to the spectrum width. In fact, the function cost is strongly dependent from any variation of the FWHM even if due to small edges deformation of the grating (to see FWHM in the Equation (12)).

### 4.2. Draw Tower Grating Arrays

All DTG arrays have been modelled setting 120 fringes per sub-grating with a wavelength resolution of 0.02 nm. Different spectral domains have been chosen in order to minimize the total number of considered wavelength during the simulation reducing computational time. Respect to the procedure adopted for CFGB, apodization profile identification is not required.

#### 4.2.1. Spatial Continuity and Spectral Discontinuity—*SpaC/SpeD*

This configuration is the most common one, as it is usually used for FBG sensor wavelength division multiplexing. It is the only configuration for which two approaches are possible for strain reconstruction.

**Figure 13.**
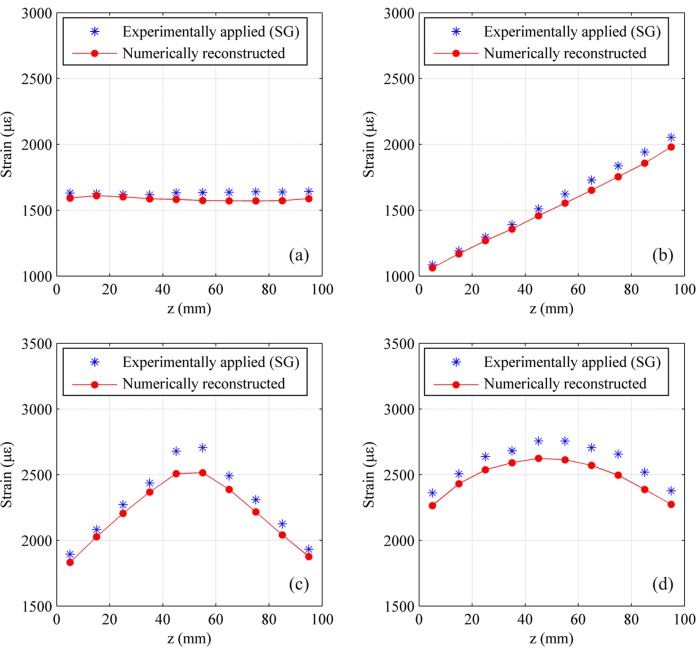
DTG array in SPAtial Continuity and SPEctral Discontinuity (*SpaC/SpeD*) configuration: strain reconstruction via individual peak tracking. Uniform strain test case (**a**); Linear strain with gradient change test case (**b**); Linear strain with gradient change test case (**c**) and Quasi quadratic strain test case (**d**).

The first is individual peak centre wavelength tracking, and results for the all the test cases are shown in Figure 13. As mentioned above, this technique allows real-time monitoring, since peak tracking can be performed via optical interrogators, avoiding time-consuming spectral analysis and numerical strain reconstruction tools. Centre wavelength tracking however does not take into account reflection spectrum shape modification, and consequently local strain gradients.

The second technique, on the other hand, involves the use of the strain reconstruction presented in Section 2. This technique, even if not applicable for real-time monitoring, allows a better description of the strain profile. For this spectral configuration however it has not been possible to use this technique due to convergence problems for the optimization algorithm.

Owing to the spectral shape of the *SpaC/SpeD* configuration, the cost function defined in Equation (12) presents strong local minima when only a fraction of the simulated peaks corresponds to the simulated ones.

#### 4.2.2. Spatial Continuity and Spectral Continuity—*SpaC/SpeC*

**Figure 14.**
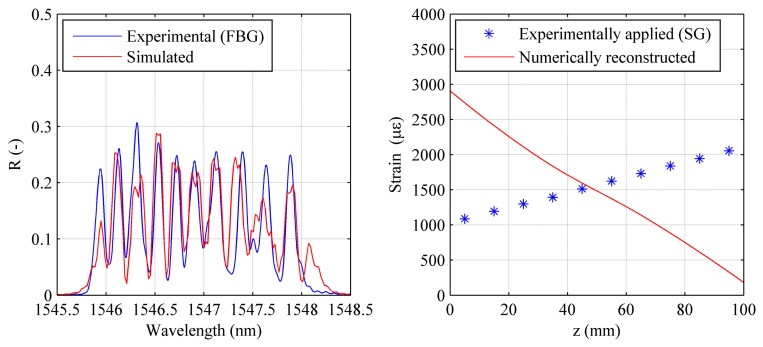
DTG array in SPAtial Continuity and SPEctral Continuity (*SpaC/SpeC*) configuration: numerically reconstructed strain profile compared with the experimentally applied one (**right**) and corresponding spectra (**left**). Linear strain test case at a maximum level of 2000 με.

Since this configuration has a continuous spectrum, the only possible approach is numerical strain reconstruction. Even in this case, numerical reconstruction has proven unsuccessful due to local minima. Such minima are caused by the lack of separation between individual peaks. As a result peaks are allowed to overlap, causing different strain profiles to have very similar spectra. For instance, in Figure 14 the experimental reflection spectrum for a linear strain profile is compared with a numerical one caused by a completely different strain profile, showing a good spectrum correspondence, which causes the local minimum problems mentioned above.

#### 4.2.3. Spatial Discontinuity and Spectral Continuity—*SpaD/SpeC*

As in the previous case, *SpaD/SpeC* configuration has a continuous spectrum, thus requiring spectral analysis and the use of the strain reconstruction program. Due to the reduced grating length, this time individual peaks are wider, leading to a more uniform reflection spectrum, more similar to the chirped grating one. This increased uniformity however is obtained at the expense of spatial continuity, causing this sensor to provide a discontinuous information.

**Figure 15.**
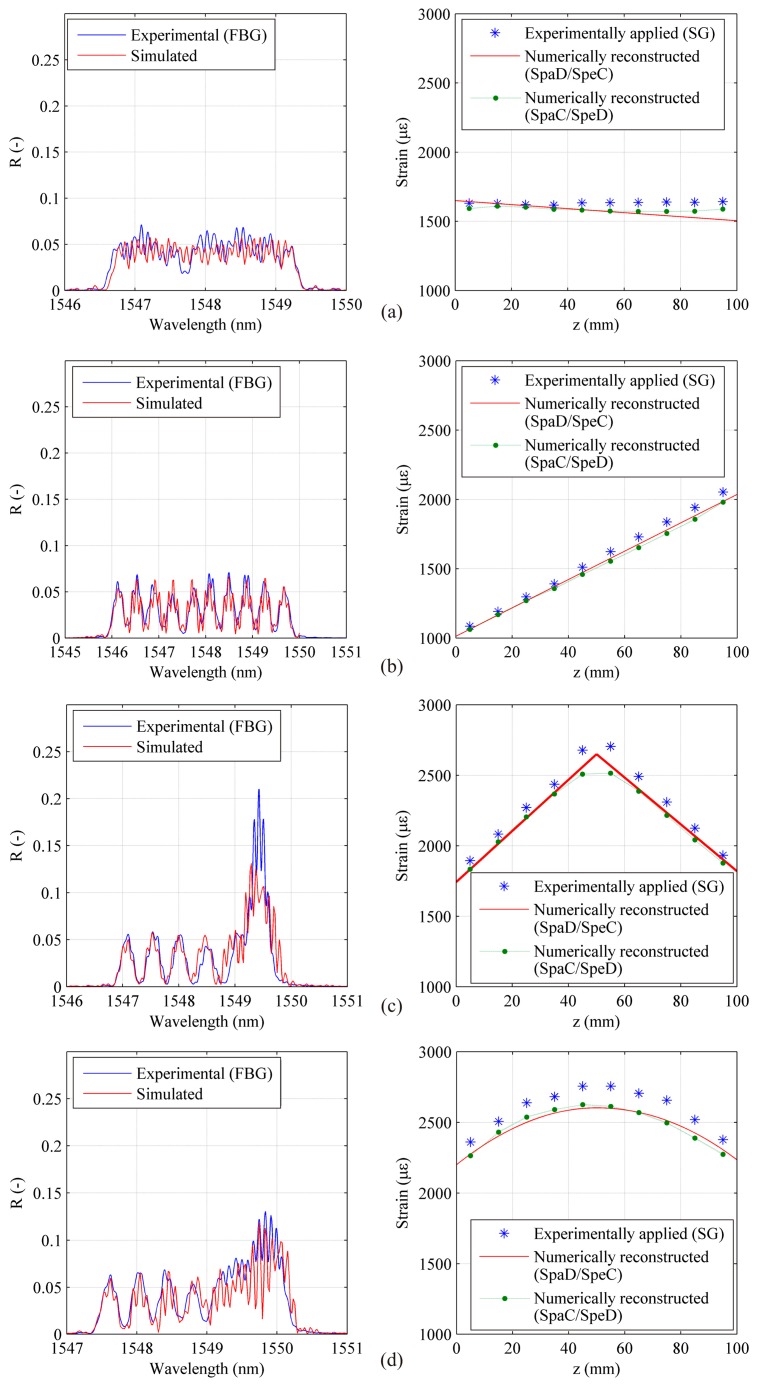
DTG array in SPAtial Disontinuity and SPEctral Continuity (*SpaD/SpeC*) configuration: numerically reconstructed strain profile compared with the experimentally applied one (**right**) and corresponding spectra (**left**). Uniform strain test case (**a**), linear strain test case at a maximum level of 2000 με (**b**), linear strain with gradient change (**c**) and quasi quadratic strain test case (**d**). Green line refers to *SpaC/SpeD* configuration previously examined.

The absence of evident individual peaks and greater spectrum uniformity make numerical reconstruction possible, as shown in Figure 15. As it can be seen in the figures for all the *SpaD/SpeC* DTG arrays the relative errors between reconstructed strain levels and SG reference ones are limited confirming the good working of this array configuration. The numerically reconstructed strain profile referred to *SpaC/SpeD* DTG arrays is also reported in the graphs in order to compare with each other the two configurations.

The curves are very similar for all test cases. An appreciable difference can be observed only in the Figure 15c where the *SpaD/SpeC* configuration is able to retrieve also the local change of the strain gradient. The computational time for full cycle analyses has been of about 150–300 min depending on the parameter setup.

## 5. Conclusions

Starting from the implementation of a numerical strain reconstruction program, this paper has shown how this can be used together with different types of FBG sensors for the development of a complete SHM system. The coupling between the developed program and a chirped grating sensor yielded excellent results for simple strain profiles applied to the sensor. However the post-processing activity is extremely time consuming, making such sensors inapplicable to real-time monitoring. As for the DTG arrays, three configurations have been tested under identical load conditions. The *SpaC/SpeD* configuration has been efficiently used to identify applied strain via direct peak tracking but numerical reconstruction proved unsuccessful due to optimization algorithm convergence issues. Similar problems have arisen for the *SpaC/SpeC*, but this configuration did not allow direct peak tracking due to the low wavelength separation between individual sensors. Finally for the *SpaD/SpeC* it has been possible to successfully perform the numerical reconstruction procedure for every load case. Through these numerous tests it has been shown that numerical strain reconstruction tools can be profitably applied to individual and arrays of FBG sensors having the suitable spectral characteristics.

## Figures and Tables

**Table 1.** Physical and optical characteristics of chirped sensors.

**Sensor Type**	**Chirped**
Total Length *L* (mm)	30
Bragg wavelength *λ_B_* (nm)	1,549.85
FWHM (nm)	45
Chirped rate (nm/mm)	1.5
Number of fringes (-)	56,520

**Table 2.** Geometrical characteristics of the specimens for chirped testing.

**Test Cases**	**Uniform**	**Linear**	**Linear with Gradient Change**
Material	Al 7075	Al 7075	Al 7075
Length (mm)	260	260	220
Width (mm)	50	50	50
Thickness (mm)	5	5	4
Number of SG	2	2	7

**Table 3.** Physical and optical characteristics of DTG arrays.

**Array Configuration**	**SpaC/SpeD**	**SpaC/SpeC**	**SpaD/SpeC**
Total Length *L* (mm)	100	100	100
Number of FBG (-)	10	10	10
FBGs Length (mm)	10	10	3
FBGs Pitch (mm)	10	10	10
FWHM (nm)	-	0.9	2.37
Chirped rate (nm/mm)	-	0.009	0.0237

**Table 4.** Geometrical characteristics of the specimen for DTG arrays.

**Test Cases**	**All**
Material	Al 7075
Length (mm)	500
Width (mm)	50
Thickness (mm)	5
Number of SG	10
Number of DTG arrays	3

## Nomenclature

CFBG: Chirped Fibre Bragg Gratings
CMT: Coupled Mode Theory
DTG: Draw Tower Grating
FBG: Fiber Bragg Grating
FWHM: Full Width at Half Maximum
HUMS: Health and Usage Monitoring Systems
SG: Strain Gauge
SHM: Structural Health Monitoring
SpaC/SpeC: SPAtial Continuity and SPEctral Continuity
SpaC/SpeD: SPAtial Continuity and SPEctral Discontinuity
SpaD/SpeC: SPAtial Discontinuity and SPEctral Continuity
TMM: Transfer Matrix Method
